# Mental health inequality between urban and rural youth under COVID-19 from survey data from China

**DOI:** 10.3389/fpubh.2024.1389765

**Published:** 2024-05-17

**Authors:** Yang Xiao

**Affiliations:** School of Philosophy, Shaanxi Normal University, Xi’an, China

**Keywords:** mental health, socioeconomic status, social capital, urban–rural differences, COVID-19

## Abstract

**Background:**

While health inequality has been the focus of past scholarly discussions, COVID-19’s outbreak and spread have provided a new arena for discussing health inequality, particularly in the context of urban–rural disparities in China. This paper explores the impact of COVID-19 on urban–rural health inequality, and the roles played by socioeconomic status and social capital.

**Methods:**

A cross-sectional observational collected data on demographics, mental health, socioeconomic status, and social capital. An online survey was administered from August 27 to August 30, 2020, and, 1936 valid samples were received. Mental health was measured using the Brief Symptom Inventory (BSI-18). This study applied the ordinary least squares regression (OLS) model, and data analysis was performed using STATA.

**Results:**

There were 1936 participants, with an equal distribution of genders. Multiple regression analysis showed that the mental health levels of rural youth were superior to those of urban youth (*p* = 0.049), especially when the epidemic was not severe (*p* = 0.013). Socioeconomic status had a significant positive promotion effect on mental health (*p* = 0.008), but the interaction effect between socioeconomic status and the urban–rural divide indicated that the promotion effect of socioeconomic status on the mental health of urban youth was greater than that of rural youth (*p* = 0.04). Social capital had a significant positive promotion effect on mental health (*p* = 0.000), and the interaction effect indicated that this promoting effect did not differ between urban and rural areas (*p* > 0.05).

## 1 Introduction

Studies on disasters have shown that besides harming to people’s lives and property, disasters can have long-term effects on people’s mental health. However, these effects are not uniform or randomly distributed (1, 2). Large-scale socio-epidemiological investigations have also revealed a highly stable inverse relationship between socioeconomic status and mental disorders (3–5). Scholars refer to this phenomenon as health inequality (6–10). As a global public health emergency, the outbreak and spread of COVID-19 have provided a new arena for discussing health inequalities.

China’s unique urban–rural dual system has led to a large gap between rural and urban areas in levels of economic development, medical resources, and social security (11, 12). Residents’ health shows a high degree of urban–rural inequality (13). COVID-19, a highly contagious disease, is spread through person-to-person contact and asymptomatic transmission. Did the outbreak and spread of COVID-19 widen or narrow the health inequalities between urban and rural areas?’.

The relationship between socioeconomic status and mental health is considered another form of health inequality (14). Research has indicated differences in the health effects of socioeconomic status between urban and rural areas (15, 16). What impact did the outbreak and spread of COVID-19 have on the differential effects of socioeconomic status on the mental health of urban and rural residents?

Social capital, as a key factor influencing health inequality, has become a focal point for scholars (17, 18). However, epidemic prevention measures such as home quarantine and maintaining social distance have confined individuals to isolated spaces, disrupting interpersonal connections. Communication has been limited to online interactions, strengthening family-centered traditional social relationships and forming new forms of epidemic social capital (19). How will this new form of epidemic social capital affect health inequality among urban and rural youth?

This paper analyzes the above three questions using online survey data.

## 2 Literature review and hypotheses

### 2.1 Mental health disparities between urban and rural youth

The classic stress exposure mechanism posits that there are structural differences in the extent of exposure to stressors, with lower socioeconomic status groups subjected to more stress due to harsh living and working environments (20, 21). It is this structural difference that leads to health inequality. The direction of the stress exposure mechanism needs to be reconsidered when applying a unique stressor like the COVID-19 pandemic, because the large-scale population concentration and high mobility in urban areas exacerbate virus transmission, whereas rural areas have lower population density and more dispersed living conditions that, are less conducive to virus spread. Therefore, compared to rural youth, urban youth are more exposed to stressors from the COVID-19 pandemic, potentially resulting in lower levels of psychological well-being. Hence, the following research hypothesis is proposed:

*Hypothesis 1*: urban youth’s mental health is lower than rural youth.

### 2.2 The urban–rural difference in the effect of socioeconomic status

The urban–rural difference in the effect of socioeconomic status on health inequality has received extensive support in domestic and international research. Further studies indicate that the effect of socioeconomic status on health can be influenced by the level of national or regional development (13). The effect of socioeconomic status on health is smaller in regions with higher economic income levels, while the effect is greater in regions with lower income levels (15, 16). This is a manifestation of the theory of resource substitution, where the absence of one resource makes another resource more valuable (14, 22). Since China’s unique urban–rural system results in significant disparities between urban and rural areas in terms of medical health security and accessibility to medical services (11, 12), the lack of public resources for rural youth during the pandemic made them more reliant on the socioeconomic status resources they possess. Hence, the following research hypothesis is proposed:

*Hypothesis 2*: the effect of socioeconomic status on the mental health of rural youth is greater than that of urban youth.

### 2.3 The urban–rural difference in the effect of social capital

Bian and his colleagues conducted a specific study on the relationship between social capital and mental health during the COVID-19 pandemic, pointing out that both bonding social capital and bridging social capital can enhance people’s ability to resist risks, thereby improving their health (19, 23, 24). Compared to urban areas, the urban–rural dual structure results in poorer institutional development for health protection in rural areas (such as medical and health facilities, medical insurance systems, etc.). According to the theory of resource substitution, when rural youth are disadvantaged in terms of access to public medical resources, they will rely more on social capital resources. Thus, social capital resources are likely to have a greater impact on the mental health of rural youth. Therefore, the following research hypothesis is proposed:

*Hypothesis 3*: the effect of social capital on the mental health of rural youth is greater than that of urban youth.

## 3 Method

### 3.1 Data

An online survey was carried out via the data collection platform “Researcher,” a professional online survey tool developed by Hanyi Data Technology (Shenzhen) Co., Ltd. Its sample frame has 30,000 of the most active WeChat users in Chin’s urban and rural areas, with an average age of 30, and has been applied in several scientific research projects. The survey was continuously pushed online from August 27 to August 30, 2020. The questionnaire was randomly sent according to a male-to-female ratio of 1:1. The respondents received and answered the questionnaires through WeChat software on their mobile terminals. To ensure data quality, we set each IP address to submit only one questionnaire, and utilized technical means to screen out invalid questionnaires, such as robot answering. We received 1936 valid samples. The samples were distributed in 32 provinces, autonomous regions and municipalities directly under the Central Government, except for Tibet and Macau.

### 3.2 Measures

#### 3.2.1 Personal information

We gathered personal information such as gender (male and female), age, marital status (married and unmarried), religious belief (yes and no), residence, and Chinese Communist Party (CCP) membership. For residence, respondents were asked if they lived in an urban or rural community at the time of the survey. In Western countries, political party affiliation is a personal choice and related to social-political values. In China, research has shown that CCP members are a selective group of elites and quasi-elites.

#### 3.2.2 Mental health

Participants’ mental health during the epidemic was assessed using the Brief Symptom Inventory-18 (BSI-18). The BSI-18 is an 18-item self-report scale of statements that participants respond to based on their level of distress over the preceding seven days (a 5-point Likert-like scale from 0, not at all, to 4, extremely). For convenience, this study converted scores. The higher the final score, the better the mental health.

#### 3.2.3 Socioeconomic status

Many scholars have measured socioeconomic status using a combination of income, education and occupation, which this study also uses. Income is the annual household income of the respondent in the past year; education is the number of years of the respondent’s education; and occupation is divided into five levels from low to high. Factor analysis was done on these three variables, and a common factor representing social and economic status was extracted. The factor contribution rate was 52.83%. For convenience, this study transformed it into a score with a range of values from 1 to 100 (25). The higher the score, the higher the socioeconomic status.

#### 3.2.4 Social capital

Social capital, including bonding social capital and bridging social capital (19), was extracted using five questions from the questionnaire: harmony of marital relationships, harmony with other family members, the frequency of online communication with relatives and friends, the frequency of internet usage, and the number of people contacted daily. Two common factors were extracted, with a cumulative contribution rate of 60.54%. The first common factor represents bonding social capital, which is composed of harmony of marital relationships and harmony with other family members. A higher value of this variable indicates a higher level of personal social engagement. The second common factor represents bridging social capital, composed of harmony with other family members, the frequency of online communication with relatives and friends, the frequency of internet usage, and the number of people contacted daily. The higher this variable’s value, the greater the extent of communication with other groups. For convenience, the values of both common factors are transformed to a range of 1–100, where higher scores represent more social capital.

#### 3.2.5 COVID-19 severity

COVID-19 is measured by whether infected or dead people are in the community. A community without infection and death is considered not serious with a value of 0. The presence of an infected person or death in the community is considered serious and assigned a value of 1.

### 3.3 Data analysis

Data analysis was performed using STATA version 17. Continuous variables were summarized as means ± standard deviation (SD); while categorical variables were summarized using frequency and percentages. Mental health is a continuous variable, and this paper mainly uses the ordinary least squares regression (OLS) model to estimate it. Firstly, based on controlling demographic variables, a multiple linear regression model was constructed to compare the differences between urban and rural youth mental health. According to epidemic severity, a sub-sample regression analysis was conducted to discuss the differences between urban and rural youth mental health. Finally, an interaction model of residence and socioeconomic status, residence and social capital was further established to test separately whether there were differences between urban and rural in the effects of socioeconomic status and social capital on youth mental health.

## 4 Results

### 4.1 Demographic characteristics and background

Our analysis was based on 1,936 validated questionnaires. The average age of the sample was 27 years, with 49% of the male sample and 26% of the youth living in rural areas. About 34% of the respondents had a severe epidemic level in their community at the time of the survey. The mean value of the mental health level is 82.32 and the standard deviation is 9.98 (Table 1).

**Table 1:** Participant demographics.

Variable	Variable assignment	*N*	Mean value	SD
Gender	Male = 1, Female = 0	1936	0.49	0.50
Age	15–40	1936	26.71	5.73
Marital status	Married = 1, Unmarried = 0	1936	0.70	0.46
CCP membership	Yes = 1, No = 0	1936	0.17	0.38
Religious belief	Yes = 1, No = 0	1936	0.15	0.35
Mental health	26–90, The larger the value, the healthier it is	1936	82.32	9.98
Residence	Rural = 1, Urban = 0	1936	0.26	0.44
COVID-19 Severity	Severe = 1, Not severe = 0	1936	0.34	0.47
Socioeconomic status	1–100, The larger the value the higher the socioeconomic status	1936	58.43	16.15
Bonding social capital	1–100, The larger the value, the more bonding social capital	1936	65.79	15.46
Bridging social capital	1–100, The larger the value, the more bridging social capital	1936	81.05	8.63

### 4.2 The impact of the COVID-19 on urban and rural youth mental health

Table 2 shows the analysis results of the multiple linear regression model for urban and rural youth mental health. The variable coefficient of the epidemic’s severity was negative and significant in the full-sample model, which means that the mental health level of youth in areas with severe epidemics was lower than that of youth in areas with less severe epidemics. The rural youth level of mental health was 0.89 units higher than urban youth, indicating the unique stressor of the COVID-19 pandemic has a significantly more negative impact on the mental health of urban youth compared to rural youth. This negative effect diminishes the resource advantage of urban youth in coping with the COVID-19 pandemic.

**Table 2:** Multiple linear regression analysis of the impact of the COVID-19 on urban and rural youth mental health.

	Full sample		Sample of less severe epidemic		Sample of severe epidemic	
	*B*	*p*-value	*B*	*p*-value	*B*	*p*-value
COVID-19 Severity	−3.35 (0.474)	0.000^***^	/	/	/	/
Residence	0.89 (0.501)	0.049^*^	1.38 (0.557)	0.013^*^	−0.93 (1.141)	0.416
Gender	0.03 (0.444)	0.947	−0.17 (0.493)	0.734	0.58 (0.882)	0.511
Age	0.16 (0.043)	0.000^***^	0.15 (0.047)	0.002^**^	0.22 (0.089)	0.015^*^
Marital status	1.08 (0.534)	0.042^*^	2.13 (0.573)	0.000^***^	−1.92 (1.156)	0.097
Religious belief	−1.93 (0.636)	0.003^**^	−0.17 (0.768)	0.827	−4.12 (1.111)	0.000
CCP member	0.86 (0.603)	0.153	0.71 (0.701)	0.313	1.45 (1.107)	0.192
Satisfaction with the control of the epidemic	3.94 (0.789)	0.000^***^	3.64 (0.867)	0.000^***^	5.29 (1.617)	0.001^**^
Constant	74.76 (1.364)	0.000^***^	74.37 (1.467)	0.000^***^	71.24 (2.843)	0.000^***^
*N*	1936		1,280		656	
Adj-*R*^2^	0.060		0.050		0.048	

Standard error in parentheses; *p < 0.05, **p < 0.01, and ***p < 0.001. Dummy variables: Gender (male = 1, female = 0); Residence (rural = 1, urban = 0); Marital status (Married = 1, unmarried = 0); and the remaining dummy variables (yes = 1, no = 0).

Did rural youth have higher levels of mental health than urban youth at any time during the pandemic? To answer this question, a sub-sample analysis was conducted based on the severity of the epidemic in the community where the youth lived. When the pandemic was less severe, the perceived pressure of the COVID-19 pandemic was higher among urban youth than rural youth, leading to higher mental health levels among rural youth compared to urban youth. When the pandemic was more severe, there was no longer a difference in the mental health levels between urban and rural youth.

### 4.3 Influence of socioeconomic status and social capital on the mental health level of youth: urban and rural differences

Table 3 reports the results of a multiple linear regression of the effects of socioeconomic status and social capital on youth mental health during the COVID-19 epidemic. In the full model, socioeconomic status variable shows that for every one-unit increase in socioeconomic status, there was a 0.04-unit improvement in the mental health of urban and rural youth. Both variables of social capital-bonding social capital and bridging social capital-contributed positively to the mental health of urban and rural youth. The regression coefficient of the residence variable was 1.32 and passed the significance test, indicating the mental health of rural youths was better than that of urban youths after controlling for other variables.

**Table 3:** Urban–rural differences in the impact of socioeconomic status and social capital on youth mental health levels.

	Full Model		Interaction model between residence and socioeconomic status		Interaction model between residence and social capital	
	*B*	*p*-value	*B*	*p*-value	*B*	*p*-value
Socioeconomic status	0.04 (0.015)	0.008^**^	0.07^***^(0.018)	0.000^***^	0.04 (0.015)	0.008^**^
Bonding social capital	0.19 (0.016)	0.000^***^	0.19^***^(0.016)	0.000^***^	0.19 (0.018)	0.000^***^
Bridging social capital	0.11 (0.025)	0.000^***^	0.11^***^(0.025)	0.000^***^	0.12 (0.031)	0.000^***^
Residence	1.32 (0.512)	0.010^*^	6.02 (1.693)	0.000^***^	6.15 (4.550)	0.177
Residence* socioeconomic status	/	/	−0.09 (0.030)	0.004^**^	/	/
Residence*Bonding social capital	/	/	/	/	−0.03 (0.031)	0.390
Residence*Bridging social capital	/	/	/	/	−0.04 (0.052)	0.462
COVID-19 Severity	−3.29 (0.457)	0.000^***^	−3.28 (0.456)	0.000^***^	−3.31 (0.458)	0.000^***^
Gender	−0.38 (0.428)	0.369	−0.43 (0.428)	0.315	−0.39 (0.428)	0.368
Age	0.09 (0.044)	0.030^*^	0.09 (0.044)	0.036^*^	0.09 (0.044)	0.031^*^
Marital status	−1.56 (0.571)	0.006^**^	−1.56 (0.570)	0.006^**^	−1.56 (0.571)	0.006^**^
Religious belief	−2.10 (0.611)	0.001^**^	−2.11 (0.610)	0.001^**^	−2.10 (0.612)	0.001^**^
CCP member	−0.09 (0.593)	0.885	−0.18 (0.592)	0.765	−0.12 (0.594)	0.845
Satisfaction with the control of the epidemic	1.68 (0.778)	0.031^*^	1.69 (0.777)	0.030^*^	1.70 (0.779)	0.029^*^
Constant	57.22 (2.465)	0.000^***^	55.51 (2.529)	0.000^***^	55.44 (2.982)	0.000^***^
*N*	1936		1936		1936	
Adj-*R*^2^	0.130		0.133		0.135	

Standard errors are in brackets; *p < 0.05, **p < 0.01, and ***p < 0.001. Dummy variables: Gender (male = 1, female = 0); Residence (rural = 1, urban = 0); Marital status (Married = 1, unmarried = 0); and the remaining dummy variables (yes = 1, no = 0).

With age increase, the mental health level of urban and rural youth rose. The mental health level of married youth was lower than that of unmarried youth. The epidemic control satisfaction variable shows that satisfaction with local epidemic control measures helped urban and rural youth improve their mental health.

To test the difference between urban and rural in the effectiveness of two supportive resources, socioeconomic status and social capital, on youth health, Table 3 continues constructing the interaction model between residence and socioeconomic status and the interaction model between residence and social capital. The reason for constructing an interaction model instead of directly comparing coefficients from separate urban and rural subsamples is to avoid sample selection bias caused by population mobility between urban and rural areas and estimation errors resulting from differences in sample sizes (26).

Firstly, the results of the interaction model between residence and socioeconomic status are shown in Table 3. The interaction term between residence and socioeconomic status is negative and passes the significance test, indicating that the contribution of socioeconomic status to rural youth mental health is less than that of urban youth. It indicates that during the pandemic, urban youth have become more dependent on their socioeconomic status compared to normal social conditions. This may be attributed to the nature of COVID-19 as a highly contagious disease. In urban areas, both high and low-status youth need to venture outside, interact with people, and may even enter the labor market to survive, thereby facing the risk of contracting COVID-19. The higher the economic status of urban youth, the more likely they are to have access to protective and subsistence materials, and the more likely they are to be employed in jobs with a low risk of unemployment and high returns, and thus better protected against the risk of COVID-19. On the other hand, urban youth with lower socioeconomic status experience greater pressure during the pandemic and are more prone to developing mental health issues. Rural youth’s income sources include agricultural income and non-agricultural income. Agricultural income mainly comes from contact with land and crops, but less contact with people, so the possibility of contracting COVID-19 is low. Non-agricultural income is more obtained through non-agricultural work, which inevitably involves contact with people and leads to a higher possibility of contracting COVID-19. Those groups in rural areas with high socioeconomic status are mostly those with non-agricultural income and are more likely to be infected with COVID-19 than those with low socioeconomic status. So, while high social status in rural areas increases the ability of rural youth to cope with COVID-19, it is also high social status that puts them at higher risk of contracting COVID-19.

Then there is also the interaction model between residence and social capital. It can be seen that the interaction term coefficient between residence and bonding social capital is negative but fails the significance test, and the interaction term coefficient between residence and bridging social capital is negative and also fails the significance test, which means that there was no difference between urban and rural areas regarding the contribution of bonding and bridging social capital to youth mental health.

## 5 Discussion

The COVID-19 pandemic created a public health crisis on an unprecedented scale, affecting everyone psychologically, although to different extents. This study used WeChat user survey data to analyze the mental health status of young people under the age of 40 and explored the role played by China’s unique urban–rural dual system in this crisis.

We found the mental health of rural youth was better than that of urban youth during the COVID-19 pandemic. Although this contradicts previous research conclusions before the epidemic, it confirms our research hypothesis 1. This indicates that the negative impact of this stressor, COVID-19, on the mental health of urban youth is significantly higher than that of rural youth. This negative impact undermines the resource advantage of urban youth in coping with the COVID-19 pandemic, mainly because rural areas have lower population density and more dispersed living conditions. Virus transmission is less likely in such conditions than in large-scale population gatherings and high mobility in urban areas. However, this situation Icured in communities where the epidemic was not severe. When an epidemic becomes severe, there is no longer a difference in the mental health levels between urban and rural youth.

During the COVID-19 period, we found that the promotion effect of socioeconomic status on the mental health of urban youth was greater than that of rural youth. This is a provocative finding because the resource substitution theory posits that urban youth have better access to public resources, and the effect of socioeconomic status on their mental health should be weaker than that of rural youth. However, the reality was quite the opposite. This may be determined by the characteristics of COVID-19 as a large-scale infectious disease. Some research indicates that while maintaining social distancing is important to prevent virus transmission, it is difficult to implement in some occupations (27) there are mainly concentrated in urban areas. In urban areas, high-status and low-status youth face the same risk of virus infection, but high-status youth resist the risk of virus infection better than low-status youth (28). Rural areas present a different scenario. The income sources of rural youth include both agricultural and non-agricultural income. Agricultural income is mainly related to land and crops, less contact with people, and lower likelihood of contracting COVID-19, while non-agricultural income is obtained through non-agricultural work, inevitably involving contact with people and thus a higher likelihood of COVID-19 infection. In rural areas, those with high socioeconomic status are often groups with non-agricultural income who (29), are more likely to contract COVID-19 than those with low socioeconomic status. Therefore, in rural areas, high socioeconomic status may enhance the ability of rural youth to cope with COVID-19, but it also exposes them to a higher risk of contracting the virus due to their high socioeconomic status.

Both variables of social capital, bonding social capital and bridging social capital, have a positive promotion effect on the mental health of urban and rural youth. Bonding social capital includes relationships such as marital and familial ties, measuring the closeness of interaction between urban and rural youth and their core network members during the epidemic period. This close connectivity is an important source of social support for urban and rural youth, enhancing their levels of mental health. Bridging social capital includes acquaintances outside intimate circles, online networks, and avenues through which people exchange information, constituting a network for individuals to obtain external support. This helps urban and rural youth maintain connections with the outside world, enabling them to promptly receive diverse information about the COVID-19 epidemic, further promoting mental health. Additionally, the promotion effect of social capital on urban and rural youth mental health does not differ. Despite urban areas being markedly superior to rural areas in terms of medical and health facilities and healthcare systems, urban youth did not rely less on social capital resources during the epidemic period simply because they have more public resources than rural youth. This partly indicates that advantaged groups do not weaken their dependence on a particular resource despite having access to multiple resources when faced with risks.

The above findings imply that although the impact of the COVID-19 pandemic on the mental health of urban and rural youth still follows the logic of health inequality, meaning that during the pandemic, groups with higher socioeconomic status have better mental health. However, when considering urban and rural areas separately, the mental health of urban youth is not higher than that of rural youth, especially in regions with less severe local epidemic. This is primarily due to the large-scale population aggregation and high mobility in urban areas, which intensify the spread of the COVID-19 virus, resulting in higher levels of risk for urban youth, particularly those with lower socioeconomic status. This highlights the need to pay attention to the mental health of urban youth with lower socioeconomic status during the pandemic.

In addition, bonding social capital, characterized by the close interaction of a core relationship circle, and bridging social capital, mainly characterized by the transmission of heterogeneous information, play an important role in enhancing the level of mental health of urban and rural youth during an epidemic. Therefore, the cultivation of social capital of urban and rural youth should be strengthened under the current situation of normalization of epidemic prevention and control.

This study compares the differences in the mental health of urban and rural youth during the COVID-19 epidemic, and analyses the differences in the effects of two factors, socioeconomic status and social capital, on the mental health of youth between urban and rural areas, but there are some shortcomings in this study. Firstly, Due to the limitations of online surveys, all the data obtained in this study are from WeChat users, which may lead to sample bias as it does not include young individuals who do not use WeChat. This raises concerns about the generalizability of the research findings. Secondly, the study found that urban youth had lower mental health compared to rural youth due to higher exposure to COVID-19 pandemic-related stress during the pandemic. However, stress exposure remains theoretical in the paper, and more rigorous data is needed to support this claim. Lastly, the study revealed that the effect of socioeconomic status on the mental health of urban youth is greater than that of rural youth, while the effect of social capital on urban and rural youths’ mental health does not differ. The inconsistent performance of socioeconomic status and social capital as influencing factors on the mental health of urban and rural youth requires further analysis.

## 6 Conclusion

Health inequality that altered between urban and rural residents during COVID-19 was examined, with rural youth exhibiting better levels of mental health than urban youth in areas where the epidemic was not severe. The impact of socioeconomic status and social capital on urban–rural health inequality was examined and found not to follow the resource substitution theory, i.e., advantaged groups do not weaken their dependence on a particular resource despite having access to multiple resources when encountering risks.

## Data availability statement

The raw data supporting the conclusions of this article will be made available by the authors, without undue reservation.

## Ethics statement

The studies involving humans were approved by the Scientific and Ethical Review Committee of Shaanxi Normal University (No. 202002001). The studies were conducted in accordance with the local legislation and institutional requirements. The participants provided their written informed consent to participate in this study. Written informed consent was obtained from the individual(s) for the publication of any potentially identifiable images or data included in this article.

## Author contributions

YX: Writing – original draft, Writing – review & editing.
